# Influencing factors of LDCT recommendation by physicians in Sichuan Province, China

**DOI:** 10.3389/fonc.2022.1049096

**Published:** 2023-01-04

**Authors:** Ruicen Li, Qi Chai, Feng Chen, Qing Liu, Hong Zhang

**Affiliations:** ^1^ Department of Health Management Center, West China Hospital, Sichuan University, Chengdu, Sichuan, China; ^2^ Department of Industrial Engineering and Engineering Management, College of Business, Sichuan University, Chengdu, Sichuan, China; ^3^ Integrated Care Management Center, West China Hospital, Sichuan University, Chengdu, Sichuan, China

**Keywords:** influencing factors, low-dose spiral CT, lung cancer screening, recommending willingness, perception of barriers

## Abstract

The study aimed to investigate the influencing factors of physicians in recommending low-dose computed tomography (LDCT) for lung cancer screening to high-risk groups. A total of 1767 participants with good knowledge of LDCT were included in a cross-sectional study. Data about physicians’ demographics, perception of barriers on LDCT screening, medical conditions for practicing medicine and the behavior of recommending LDCT were collected by a questionnaire. Physicians who care about the transportation convenience of patients were less likely to recommend LDCT (OR 0.568, 95% CI (0.423 to 0.763), *p* < 0.05). The physicians who considered LDCT expensive, recommended LDCT less than others (OR 0.308, 95% CI (0.186 to 0.510), *p* < 0.05). The false positive rate of LDCT can decrease the possibility of physicians’ recommending (OR 0.542, 95% CI (0.387 to 0.758), *p* < 0.05). The physicians in oncology department and health management center were more likely to recommend LDCT (OR 2.282, 95% CI (1.557 to 3.345); OR 2.476, 95% CI (1.618 to 3.791)). The convenience of transportation, the price, and the\ false positive rate may be the main concerns among physicians on recommending LDCT to high-risk groups. The influencing factors of physicians’ recommending on LDCT was various. Information technology, government support in price and self-improvement of LDCT should be gathered together to break the barriers on physicians’ recommending on LDCT.

## 1 Introduction

Worldwide, lung cancer is currently the most common cancer, accounting for 11.6% of all cancers and the leading cause of cancer-related mortality, responsible for 18.4% of overall cancer mortality in both men and women (1). Similarly, lung cancer is the most common cancer in China (2), accounting for 20% of all cancers and 27% of all cancer-related death (3). Previous studies, such as US National Lung Screening Trial (NLST) and the German Lung cancer Screening Intervention (LUSI), have shown that lung cancer screening by low-dose computed tomography (LDCT) effectively reduces lung cancer mortality (4, 5). However, the screening benefits are largely dependent on the high-risk population’s attendance. Currently, lung cancer screening is performed in many countries, but the screening compliance is mostly lower than 60% (6, 7). In China, previous studies revealed that lung cancer screening compliance is lower, roughly reaching forty percent (8, 9).

And Since healthcare workers are often the primary source of health-related information for patients (10, 11), and the decision of lung cancer related examinations were always made by pulmonologist, so the physician especially the lung cancer related doctors are likely to play the key role in promoting the screening compliance.

Improving understanding of lung cancer screening knowledge among doctors may increase utilization of LDCT has been proved by many studies (12–14). But we found the perceptions of barriers on screening were similar among the doctors whether aware of the knowledge of LDCT (12). In other words, there is a possibility that doctors who aware of the knowledge of lung cancer will not recommend LDCT to high-risk groups. Therefore, this study is devoted to discuss the factors that influence the recommendation of LDCT to high-risk groups by physicians with good knowledge and which point has rarely been mentioned in previous studies. Understanding the factors influencing the doctors’ recommendation of LDCT can help guide future interventions aimed at promoting the screening rate.

According to some classical theory of framework, some personal factors and organizational factors were deemed to be the main factors to improve doctors’ compliance on recommending LDCT (15). And we combined with existing studies, the sociodemographic characteristics of doctors (16), the perceptions of barriers on LDCT (12) and medical conditions for practicing medicine (17, 18) may influence medical personnel’s medical behavior decision. The mechanisms of human behavior are complex, so we want to combine these factors to discuss how they influence doctors’ behavior.

## 2 Materials and methods

### 2.1 Participants

In this cross-sectional study, a convenience sample of physicians at a general hospital who were willing to participate were recruited. These physicians all came from Sichuan Province, China. The majority of them belonged to departments that were related to lung cancer, including the respiratory department, thoracic surgery department, oncology department, and health management center, a few of them from other departments. Excluding the missing date from any of the variables used in the present study, 1767 physicians remained. These physicians had all self-reported they had finished networking learning of lung cancer related knowledge from West China Hospital and had a good self-evaluation about grasping the knowledge of LDCT.

### 2.2 Survey questionnaire

The survey contained four sections:(1) The doctors’ sociodemographic characteristics, and in this study included gender, age, education, professional title, department, and experience. (2) The doctors’ perception of barriers on LDCT screening, and by combining with existing studies, we find the high price of LDCT (19), false positive rate (20, 21), frequent radiation (22, 23), and its mental impact (24) are major perceptions of barriers on LDCT recommending among doctors. So, we used the four questions “Do you consider the price of low-dose spiral CT expensive?”, “Do you think the false positive rate of LDCT is high?”, “Do you think radiation exposure to LDCT is unacceptable compared with chest X-ray, sputum, and other screening methods?” and “Do you think the mental burden imposed on patients by LDCT is unacceptable?” to measure respectively. And combining with the harsh geography in Sichuan province, we added the convenience of transportation as one more barrier, which was measured by the question “will you consider the convenience of transportation for patients to seek for medical service?”. All items were yes-no questions. (3) Medical conditions for practicing medicine in this study were defined by the availability of LDCT, and hospital level. (4) The behavior of recommending LDCT which was measured by the question “Have you recommended LDCT to high-risk groups in the past 12 months?”. The item was yes-no question.

### 2.3 Data collection

The Wenjuanxing platform, one of the leading companies in online questionnaire data collection in China, handled data collection. The survey was distributed to the physicians *via* WeChat (a popular social application in China). Each participant had the right to decide whether to participate in the study and could withdraw from the study at any time. Before the content of the questionnaire was presented, a page showing the objectives of this study and soliciting informed consent from the participants was presented. If the participants provided informed consent, they could continue to complete the questionnaire. Every questionnaire was check by two investigators for logistic errors and missing items. Questionnaires with more than 20% missing items were eliminated. Finally, 1767 complete responses were received.

### 2.4 Statistical analysis

Data provided by the questionnaires in Wenjuanxing were entered into the Microsoft Excel program and double-checked before analysis. And the data were imported into SPSS 23.0 for statistical analysis. Descriptive statistics, including frequency (n) and percentage (%) were used to express the distribution of data. The variables between the recommendation and no-recommendation groups were compared using the Chi-square method.

And then a multivariable logistic regression analysis was conducted to explore the predictors of the physicians’ recommendation of LDCT to high-risk groups. All variables with a significant association with the recommending behavior in aforementioned univariate tests and other variables had closely relationship with the recommending behavior according to previous studies were into the multivariable logistic regression model. The results of logistic regression are presented as odds ratios (OR) with 95% confidence intervals (CI). We performed Hosmer and Leeshawn’s goodness-of-fit test to identify if our model had a good fit with a *P*-value >0.05. And a two-tailed *P*<0.05 was viewed as statistically significant.

## 3 Results

### 3.1 The characteristics of participants

**Table 1** shows the result of univariable analysis. Among the 1767 physicians, 66% self-reported that they had recommended LDCT to high-risk groups in past 12 month. There were significant differences in the education level, professional title, department, working experience, availability of LDCT and the level of hospitals (*P*<0.05). However, no statistically significant differences were found in gender, and age group (*P*>0.05).

**Table 1:** Univariable analysis of the participants’ characteristics.

Characteristics	Total n (%)	Recommending n (%)	No Recommending n (%)	*P* value
Gender				
Male	935 (54.6)	605 (64.7)	330 (35.3)	0.208
Female	832 (45.4)	562 (67.5)	270 (32.5)	
Age groups (years)				
20-29	293 (16.6)	185 (63.1)	108 (36.9)	0.711
30-39	814 (46.1)	542 (66.6)	272 (33.4)	
40-49	454 (25.7)	304 (67.0)	150 (33.0)	
≥50	206 (11.7)	136 (66.0)	70 (34.0)	
Education level				
High school	59 (3.3)	20 (33.9)	39 (66.1)	**<0.001**
College	200 (11.3)	115 (57.5)	85 (42.5)	
Bachelor	1094 (61.9)	708 (64.7)	386 (35.3)	
Postgraduate or above	414 (23.4)	324 (78.3)	90 (21.7)	
Professional title				
Junior title	536 (30.3)	328 (61.2)	208 (38.8)	**0.003**
Intermediate title	773 (43.7)	512 (66.2)	261 (33.8)	
Senior titles	458 (25.9)	327 (71.4)	131 (28.6)	
Department				
Other departments	795 (45.0)	344 (43.3)	451 (56.7)	
Respiratory department	319 (18.1)	208 (65.2)	111 (34.8)	**<0.001**
Thoracic surgery department	181 (10.2)	122 (67.4)	59 (32.6)	
Oncology department	283 (16.0)	235 (83.0)	48 (17.0)	
Health management center	189 (10.7)	151 (79.9)	38 (20.1)	
Experience (year)				
<5 years	373 (21.1)	227 (60.9)	146 (39.1)	**0.047**
5 years≤…<10 years	438 (24.8)	287 (65.5)	151 (34.5)	
10years≤…<15years	393 (22.2)	262 (66.7)	131 (33.3)	
≥15years	563 (31.9)	391 (69.4)	172 (30.6)	
Availability of LDCT				
Have LDCT in hospital	1147 (64.9)	843 (73.5)	304 (26.5)	**<0.001**
No LDCT in hospital	620 (35.1)	324 (52.3)	296 (47.7)	
The level of hospitals				
Secondary hospital or below	407 (23.0)	208 (51.1)	199 (48.9)	**<0.001**
Tertiary hospital	1360 (77.0)	959 (70.5)	401 (29.5)	
**Total**	1767 (100)	1167 (66.0)	600 (34.0)	

Comparison using Chi-square test, significant value are in bold, *P* < 0.05.

### 3.2 Perception of barriers on LDCT screening among physicians

**Table 2** shows the result of univariate analysis. The perception of barriers on the convenience of transportation, the price and the false positive rate were statistically significant between two groups of recommendation and no-recommendation(*P*<0.05). And the difference of two group on the radiation exposure and mental burden were statistically insignificant(*P*>0.05).

**Table 2:** The univariate analysis of physicians’ perception of barriers on recommending LDCT.

Perception of barriers on LDCT	Total n (%)	Recommending n (%)	No Recommending n (%)	*P* value
Will you consider the convenience of transportation for patients to seek for medical service?				
Yes	1220 (69.1)	762 (62.5)	458 (37.5)	**<0.001**
No	547 (30.9)	405 (74.0)	142 (26.0)	
Do you consider the price of low-dose spiral CT expensive?				
Yes	85 (4.8)	39 (45.9)	46 (54.1)	**<0.001**
No	1682 (95.2)	1128 (67.1)	554 (32.9)	
Do you think the false positive rate of LDCT is high?				
Yes	1318 (74.6)	845 (64.1)	473 (35.9)	**0.003**
No	449 (25.4)	322 (71.7)	127 (28.3)	
Do you think radiation exposure to LDCT is unacceptable compared with chest X-ray, sputum, and other screening methods?				
Yes	319 (18.1)	201 (63.0)	118 (37.0)	0.206
No	1448 (81.9)	966 (67.7)	482 (33.3)	
Do you think the mental burden imposed on patients by LDCT is unacceptable?				
Yes	60 (3.4)	36 (60.0)	24 (40.0)	0.314
No	1707 (96.6)	1131 (66.3)	576 (33.7)	
Total		1167 (66.0)	600 (44.0)	

Comparison using Chi-square test, significant value are in bold, *P* < 0.05.

### 3.3 Multiple-factor analysis

We included all variables in univariate analysis that had a significant association with the recommending behavior, and the all perception of barriers whether statistically significant or not in the univariate analysis for these barriers had been proved by many previous studies (22–24).

**Table 3** shows the convenience of transportation (OR=0.568, 95%CI=0.423-0.763), the price of LDCT screening (OR=0.308, 95%CI=0.186-0.510), and the false positive outcome (OR=0.708, 95%CI=0.521-0.961) may be physicians’ main concern on whether recommending LDCT to high-risk groups.

**Table 3:** The multivariable logistic regression for factors influencing physicians’ recommendation on LDCT to high-risk groups.

Variable	Reference	*P*-value	OR (95% CI)
Transportation			
Yes	No	<0.001	**0.568 (0.423,0.763)**
Price			
Yes	No	<0.001	**0.308 (0.186,0.510)**
False positive rate			
Yes	No	<0.001	**0.542 (0.387,0.758)**
The exposure radiation of LDCT			
Yes	No	0.053	0.708 (0.521,1.011)
Mental burden			
Yes	No	0.385	0.782 (0.448,1.363)
Education level			
College school	High school	<0.001	**3.848 (1.975,7.497)**
Bachelor		<0.001	**4.254 (2.286,7.917)**
Postgraduate or above		<0.001	**7.725 (3.950,15.107)**
Professional title			
Intermediate title	Junior title	0.412	0.880 (0.649,1.194)
Senior title		0.310	0.809 (0.537,1.218)
Department			
Respiratory department	Other departments	0.519	1.107 (0.813,1.507)
Thoracic surgery department		0.834	1.042 (0.710,1.528)
Oncology department		<0.001	**2.282 (1.557,3.345)**
Health management center		<0.001	**2.476 (1.618,3.791)**
Experience(year)			
5 years≤…<10 years	Experience<5 years	0.049	**1.401 (1.010,1.990)**
10years≤…<15years		0.017	**1.630 (1.093,2.433)**
≥15years		0.001	**2.142 (1.394,3.292)**
Availability of LDCT			
Available of LDCT	Unavailable of LDCT	<0.001	**1.712 (1.325,2.210)**
The level of hospitals			
Secondary hospital or below	Tertiary hospital	0.203	1.230 (0.894,1.691)
Constant		0.144	0.502

Significant value are in bold, *P* < 0.05.

The following factors were associated with a higher possibility of recommending LDCT to high-risk groups: higher education level [OR (95%CI):3.848(1.975,7.497),4.254(2.286,7.917), and7.725(3.950,15.107) for college school, Bachelor, and Postgraduate or above, respectively], the longer work experience [OR (95%CI): 1.401(1.010,1.990), 1.630(1.093,2.433), 2.142(1.394,3.292) for different work experience], the availability of LDCT(OR=1.712,95%CI=1.325,2.210). Compared to other departments, the physicians in Oncology department (OR=2.282,95%CI=1.557-3.345) and Health management center (OR=2.476,95%CI=1.618-3.791) were more likely to recommend LDCT to high-risk groups.

## 4 Discussion

The convenience of transportation was the factors of impact in this study. Sichuan Province, our study region, is located in the transition zone between the Qinghai-Tibet Plateau and the Middle-Lower Yangtze Plain. The northwest region of Sichuan province is mainly composed of plateaus and mountains, with an elevation of more than 4000 meters. The southwest of Sichuan province is Hengduan Mountains, with numerous high mountains and deep valleys. The hostile environment impedes normal traffic. In addition, poor resources and weak financial conditions are found in most regions of Sichuan province. Therefore, the convenience of transportation for medical services may influence the willingness of physicians in Sichuan province to recommend LDCT to high-risk groups. Modern technology may help solve this problem. Advanced 5G wireless communication can be used to gather outstanding experts nationwide for accelerating diagnosis and designing therapeutic regimens.

In China, the price of medical services is uniformly designated by the government according to different grades of hospitals. For instance, in a first-class hospital in grade 3, sputum screening costs RMB75, CXR costs RMB 130.3, and LDCT costs RMB250. The price of LDCT is significantly higher than sputum screening and CXR. LDCT screening may substantially increase national health care expenditures in less developed countries. Previous cost-effectiveness analyses also have not conclusively shown that LDCT is cost-effective (22, 25, 26). And in China lung cancer screening is usually done in an outpatient setting or in a medical examination institution. Although China nearly achieved universal health insurance coverage, the outpatient fees and the fees paid in medical examination institutions are not covered by medical insurance. Therefore, reducing the cost of LDCT or adopting certain medical subsidies for poor individuals may reduce the physicians’ perceptions of barriers on LDCT and may be helpful in improving the early diagnosis of lung cancer. And we found that the cost of LDCT screening to the patient was the also the most often-cites concern among pulmonologists abroad, although some countries has covered the cost of lung cancer screening (27).

LDCT is recognized as a highly sensitive test, which is inevitably accompanied by a high false positive rate. There were many studies had reported enough information to confirmed the high false-positive rate of LDCT screening home and abroad (20, 21, 28, 29). And in this study, the high false-positive rate was also proved to be one of the main perception of barriers on recommending LDCT among physicians in Sichuan province of China. The economic development level of Sichuan Province is not balanced, and residents in most areas are relatively poor. Moreover, due to geographical location, many residents are not convenient to travel to seek for periodically lung cancer screening. So, in addition to unnecessary tests and invasive procedures, the medical burden and the difficulty of seeking for medical service also increased by the false positive outcome among the residents in Sichuan Province. Precise lung cancer screening was desperately needed in such underdeveloped areas.

The mental burden and radiation didn’t influence physicians choose in recommending LDCT or not in this study. LDCT was introduced into China because of its low radiation compared to ordinary CT and which characteristic may be recognized and accepted by majority Chinese physicians. Reasonable and accurate case selection for screening can reduce radiation exposure and unnecessary screening (30). Compared with the serious consequences of lung cancer, the anxiety of high-risk subjects may be not important enough to influence physicians’ recommending.

Physicians in health management center and oncology department were more likely to recommend LDCT to high-risk groups. Physicians in thoracic surgery and respiratory medicine were no different in the likelihood of recommending LDCT compared with other departments. The difference may be related to the different groups they faced. The healthy people and diagnosed population were more likely in health management center and oncology department. And the patients who need to be diagnosed are more likely in Thoracic Surgery and Respiratory Medicine. The fact that low-dose CT does have an impact on the quality of the image which may influence physicians’ judgement can’t be ignored.

Limited by the weak causal inference power of cross-sectional data and given that a convenience sample was used, the generalizability of the result was limited.

## 5 Conclusion

The convenience of transportation, the price, false positive rate and the quality of image may be the main perception of barriers on recommending LDCT to high-risk groups among physicians in Sichuan province. Information technology, such as 5G wireless communication and mobile CT unit which may allow effective screening for underserved populations, government support in price and self-improvement of LDCT should be gathered together to break the barriers on physicians recommending on LDCT.

## Data availability statement

The original contributions presented in the study are included in the article/supplementary material. Further inquiries can be directed to the corresponding author.

## Ethics statement

The studies involving human participants were reviewed and approved by Ethical approval was granted by the Ethical Committee of West China Hospital, Sichuan University. The patients/participants provided their written informed consent to participate in this study.

## Author contributions

RL & QC designed the study, these authors contributed equally to this work and should be considered co-first authors. RL, QC, QL and HZ collected, analyzed data. FC, RL and QC wrote the manuscript. All authors contributed to the article and approved the submitted version.
